# Twelve-year mortality in adults initiating antiretroviral therapy in South Africa

**DOI:** 10.7448/IAS.20.1.21902

**Published:** 2017-09-25

**Authors:** Morna Cornell, Leigh F. Johnson, Robin Wood, Frank Tanser, Matthew P. Fox, Hans Prozesky, Michael Schomaker, Matthias Egger, Mary-Ann Davies, Andrew Boulle, for the International Epidemiology Databases to Evaluate AIDS-Southern Africa collaboration

**Affiliations:** ^a^ Centre for Infectious Disease Epidemiology & Research, School of Public Health & Family Medicine, University of Cape Town, Cape Town, South Africa; ^b^ Division of Epidemiology and Biostatistics, School of Public Health & Family Medicine, University of Cape Town, Cape Town, South Africa; ^c^ The Desmond Tutu HIV Centre, Institute for Infectious Disease & Molecular Medicine, University of Cape Town, Cape Town, South Africa; ^d^ Africa Centre for Health and Population Studies, University of KwaZulu-Natal, Mtubatuba, South Africa; ^e^ Health Economics & Epidemiology Research Office, Department of Internal Medicine, School of Clinical Medicine, Faculty of Health Sciences, University of the Witwatersrand, Johannesburg, South Africa; ^f^ Departments of Epidemiology & Global Health, Boston University School of Public Health, Boston, MA, USA; ^g^ Division of Infectious Diseases, Department of Medicine, University of Stellenbosch and Tygerberg Academic Hospital, Cape Town, South Africa; ^h^ Division of International and Environmental Health, Institute of Social and Preventive Medicine (ISPM), University of Bern, Bern, Switzerland; ^i^ Health Impact Assessment, Provincial Government of the Western Cape, Western Cape, South Africa

**Keywords:** mortality, long-term, antiretroviral, viral suppression, gender, outcomes, Africa

## Abstract

**Introduction**: South Africa has the largest number of individuals living with HIV and the largest antiretroviral therapy (ART) programme worldwide. In September 2016, ART eligibility was extended to all 7.1 million HIV-positive South Africans. To ensure that further expansion of services does not compromise quality of care, long-term outcomes must be monitored. Few studies have reported long-term mortality in resource-constrained settings, where mortality ascertainment is challenging. Combining site records with data linked to the national vital registration system, sites in the International Epidemiology Databases to Evaluate AIDS Southern Africa collaboration can identify >95% of deaths in patients with civil identification numbers (IDs). This study used linked data to explore long-term mortality and viral suppression among adults starting ART in South Africa.

**Methods**: The study was a cohort analysis of routine data on adults with IDs starting ART 2004–2015 in five large ART cohorts. Mortality was estimated overall and by gender using the Kaplan-Meier estimator and Cox’s proportional hazards regression. Standardized mortality ratios (SMRs) were calculated by dividing observed numbers of deaths by numbers expected if patients had been HIV-negative. Viral suppression in patients with viral loads (VLs) in their last year of follow-up was the secondary outcome.

**Results**: Among 72,812 adults followed for 350,376 person years (pyrs), the crude mortality rate was 3.08 (95% CI 3.02–3.14)/100 pyrs. Patients were predominantly female (67%) and the percentage of men initiating ART did not increase. Cumulative mortality 12 years after ART initiation was 23.9% (33.4% male and 19.4% female). Mortality peaked in patients enrolling in 2007–2009 and was higher in men than women at all durations. Observed mortality rates were higher than HIV-negative mortality, decreasing with duration. By 48 months, observed mortality was close to that in the HIV-negative population, and SMRs were similar for all baseline CD4 strata. Three-quarters of patients had VLs in their last year, and 86% of these were virally suppressed.

**Conclusions**: The South African ART programme has shown a remarkable ability to initiate and manage patients successfully over 12 years, despite rapid expansion. With further scale-up, testing and initiating men on ART must be a national priority.

## Introduction

The rate of scale-up of ART services in developing countries has increased dramatically in recent years. By the end of 2015, 17 million people globally received ART, including 12 million individuals living with HIV in sub-Saharan Africa [1]. South Africa has both the largest number of individuals living with HIV and the largest ART programme worldwide. By mid-2015, 11 years after the inception of the programme, an estimated 3.39 million individuals were on ART [2]. In September 2016, South Africa implemented universal ART eligibility, extending ART eligibility to all 7.1 million HIV-positive South Africans. While the benefits of universal HIV treatment are evident, concerns that further expansion of ART programmes may compromise quality of care [3–7] make it essential to monitor trends in the mortality of patients on ART.

To date, few studies have reported long-term mortality in ART programmes in resource-constrained settings [3,8–10]. Using routine data captured by sites, we have previously reported decreasing mortality in the first year of ART in successive calendar years of ART initiation [11]. Such apparent declines in mortality may be real or could be due to deteriorating mortality ascertainment and retention in rapidly expanding ART programmes [12,13]. Indeed, as access to ART has expanded, routine data have increasingly underestimated true mortality: the proportion of deaths recorded in South African ART programmes dropped from 60% to 30% (2004–2005 compared with 2010 onwards) [13].

One innovative way of improving mortality ascertainment is through linkage of site records to vital registration systems. In South Africa, the National Population Register (NPR) records deaths of individuals with IDs and is estimated to be around 94% complete for adult deaths [13]. The inclusion of linkage data doubled cumulative all-cause mortality at four years after ART initiation in a large public sector programme in Cape Town [14], highlighting the need for accurate, recent and long-term mortality data [15]. The International Epidemiology Databases to Evaluate AIDS Southern Africa (IeDEA-SA) collaboration is uniquely placed to provide such estimates [16]. Combining site records with linkage data, sites participating in the collaboration can identify over 95% of deaths in patients with South African IDs [13].

Gender differences in mortality have been reported by many large ART programmes across Africa [17–22]. While most researchers have attributed men’s elevated mortality risk to their own poorer health-seeking behaviour, we have previously found that the observed differences in mortality on ART may be best explained by background differences in death rates, unrelated to HIV/AIDS [23]. Given men’s poorer access to HIV testing and treatment [23–28] and outcomes on treatment across Sub-Saharan Africa [17–23,29–32], long-term outcome data must also be explored by gender.

Whereas preventing HIV-associated mortality and morbidity has long been the major focus of ART programmes in high burden settings, one of the rationales for the recent change to universal access to ART is the impact of widespread viral suppression on HIV transmission. South Africa is one of the few high burden settings where routine viral load (VL) testing is available, enabling assessment of cohort-wide viral suppression.

This study used recent data to explore temporal changes in patient characteristics at enrolment and to describe long-term mortality in patients initiating ART 2004–2015 in South African sites of IeDEA-SA. Secondary objectives were to explore these trends by gender relative to the HIV-negative population, and to provide some assessment of viral suppression as a measure of non-mortality outcomes.

## Methods

### Data sources

IeDEA-SA is a regional collaboration combining routine observational data from large ART programmes in Southern Africa. In South Africa, nine adult cohorts of IeDEA-SA provide ART services in three of the most populous provinces (Gauteng, KwaZulu-Natal and Western Cape). Cohorts range in size, are predominantly government funded and follow national HIV treatment guidelines. Patients are broadly representative of HIV-positive adults accessing public sector ART in rural and urban centres [33].

This study was a cohort analysis of anonymized routine data collected prospectively from five large South African ART programmes which collect ID numbers: two in urban primary healthcare clinics (Gugulethu and Khayelitsha), two in urban hospitals (Themba Lethu and Tygerberg) and a large rural cohort of 17 primary healthcare clinics (Hlabisa).

### Eligibility criteria

We included ART-naïve adults 16–85 years old with recorded ID numbers who started ART 2004 to 2015. The analysis was limited to patients with ID numbers to ensure the best possible mortality ascertainment. Patients were followed from the date of ART initiation (regarded as baseline) to analysis closure date, which was 30 days prior to the date when site records were linked to the NPR, to make provision for delays in reporting of deaths.

### Outcomes

The primary outcome was all-cause mortality. Patients’ vital status at analysis closure was confirmed by the South African Medical Research Council through deterministic linkage with recorded deaths on the NPR. In the case of discrepancies between site- and NPR-recorded mortality, we used the site-recorded date (for deaths that were not recorded in the NPR) or the NPR date (for deaths recorded in the NPR only, and deaths recorded in both sources but with differing dates). All patients whose deaths were not recorded prior to the analysis closure date (either on the NPR or in site records), including those patients who sites classified as transferred or lost to follow-up (LTF), were recorded as alive at analysis closure.

Viral suppression among patients with VLs in their last year of follow-up was the secondary outcome. According to national guidelines, routine monitoring involves a VL test at 6 and 12 months on ART, and then every 12 months, with more frequent monitoring of patients with unsuppressed VLs [34]. We analysed the completeness of VLs in the last year of patient follow-up and the percentages of patients with suppressed VLs (measurement of less than 1000 copies/mL) by gender and calendar period of enrolment.

### Statistical analyses

Data were cleaned, coded and analysed using the Stata package (Version 13.1., College Station, TX, USA). Summary baseline characteristics (gender, age, pregnancy, weight, CD4 continuous and categorical, WHO stage and tuberculosis (TB) diagnosis) were described using proportions, medians and interquartile ranges (IQRs). We reported characteristics overall, by gender and by calendar period of ART initiation in quartiles (2004–2006, 2007–2009, 2010–2012 and 2013–2015), and compared baseline characteristics of patients with and without IDs. The number and proportion of deaths, median follow-up times and mortality rates were reported by gender. Cumulative mortality proportions and 95% confidence intervals (CIs) were presented by calendar period and time since ART initiation, overall and by gender.

Crude mortality was compared by gender using the Kaplan–Meier estimator. Cox’s proportional hazards models were used to assess crude and adjusted associations between patients’ characteristics and mortality. We adjusted models for baseline characteristics (gender, age, CD4 count, WHO stage, TB diagnosis, weight and site of ART initiation) and calendar period of ART initiation. We assumed that data were missing at random, mostly for administrative and clerical reasons, and had no reason to believe that unmeasured factors influenced the probability of missingness. We used multiple imputation [35] with chained equation methods [36] to impute missing baseline covariates 10 times: CD4 count, WHO stage, TB (yes/no) and weight. We evaluated the proportional hazard assumption using “log–log” plots for all categorical variables in the multiply imputed data sets. In all instances, the lines were approximately parallel, thus the assumption did not seem to be violated.

We calculated standardized mortality ratios (SMRs) by dividing actual numbers of deaths in IeDEA-SA patients by the numbers of deaths that would have been expected if all patients were HIV negative [37]. HIV-negative mortality rates, by age and gender, were set at the 2010 estimates from Thembisa version 2.5, a combined demographic and HIV model for South Africa [38]. We calculated actual and expected numbers of deaths separately for different baseline CD4 categories, for different times since first ART initiation, and for men and women. CIs for the SMRs were calculated on the assumption that the number of deaths in each covariate category was Poisson-distributed.

### Ethics and informed consent

All participating sites obtained ethics approval from relevant local institutions before contributing anonymized patient data to this collaborative analysis. Informed consent was not required as data were not identifiable.

## Results

Of 102,145 patients, 71% (*n* = 72,887) had IDs and were eligible for inclusion. Of these, 75 patients were excluded (date of death from the NPR prior to their recorded ART initiation date). Overall, 72,812 patients were included in the analysis.

Patients were predominantly female (*n* = 49,025, 67%) and the proportion of males initiating treatment did not increase over 12 years (Table 1). At ART initiation, men were older than women (median 38 years, IQR 33–45 vs. 33 years, IQR 28–40). The median CD4 count increased in successive calendar periods, from 101 cells/µL in 2004–2006 to 242 cells/µL in 2013–2015.

**Table 1.** Baseline characteristics of patients by calendar period of ART initiation and gender.

	**2004–2006** (*n* = 11,243)		**2007–2009** (*n* = 19,148)		**2010–2012** (*n* = 27,516)		**2013–2015** (*n* = 14,905)		**Total** (*n* = 72,812)	
**Gender, *n* (%)**										
Males	3537	(31%)	6531	(34%)	8974	(33%)	4745	(32%)	23,787	(33%)
Females	7706	(69%)	12,617	(66%)	18,542	(67%)	10,160	(68%)	49,025	(67%)
**Age (years), median (IQR)**										
Males	37	(33–43)	38	(33–45)	38	(32–45)	38	(32–44)	38	(33–45)
Females	33	(29–39)	34	(28–40)	33	(28–41)	32	(27–39)	33	(28–40)
**CD4 count (cells/µL) *n* (%)**										
Observations	9646	(86%)	17,259	(87%)	24,341	(83%)	12,788	(69%)	64,034	(82%)
Median (IQR)										
All	101	(44–163)	120	(57–179)	166	(86–243)	242	(121–344)	148	(71–227)
Males	83	(33–149)	98	(42–164)	135	(61–212)	184	(81–296)	121	(51–197)
Females	108	(51–167)	132	(66–184)	179	(100–255)	267	(147–370)	161	(84–241)
0–49										
All	2664	(28%)	3826	(22%)	3604	(15%)	1429	(11%)	11,523	(18%)
Males	1043	(34%)	1702	(29%)	1675	(21%)	674	(17%)	5094	(25%)
Females	1621	(24%)	2124	(19%)	1929	(12%)	755	(9%)	6429	(15%)
50–199										
All	5983	(62%)	10,771	(62%)	11,556	(48%)	3829	(30%)	32,139	(51%)
Males	1720	(57%)	3530	(60%)	4002	(50%)	1545	(38%)	10,797	(52%)
Females	4263	(64%)	7241	(64%)	7554	(46%)	2284	(27%)	21,342	(51%)
200–349										
All	898	(9%)	2363	(14%)	8149	(33%)	4510	(35%)	15,920	(24%)
Males	228	(8%)	611	(10%)	2018	(25%)	1348	(33%)	4205	(19%)
Females	670	(10%)	1752	(15%)	6131	(37%)	3162	(37%)	11,715	(27%)
≥350										
All	104	(1%)	306	(2%)	1046	(4%)	3026	(23%)	4482	(7%)
Males	36	(1%)	73	(1%)	249	(3%)	543	(12%)	901	(4%)
Females	68	(1%)	233	(2%)	797	(5%)	2483	(28%)	3581	(7%)
**WHO stage, *n* (%)**										
Observations	5529	(49%)	10,665	(56%)	17,264	(63%)	10,640	(72%)	44,098	(61%)
I and II										
Males	273	(16%)	689	(19%)	2133	(39%)	1620	(52%)	4715	(34%)
Females	939	(24%)	2477	(35%)	7084	(60%)	5463	(73%)	15,963	(53%)
III										
Males	930	(55%)	2149	(59%)	2366	(44%)	1172	(37%)	6617	(48%)
Females	1999	(52%)	3408	(48%)	3528	(30%)	1586	(21%)	10,521	(35%)
IV										
Males	486	(29%)	783	(22%)	911	(17%)	339	(11%)	2519	(18%)
Females	902	(23%)	1159	(16%)	1242	(11%)	460	(6%)	3763	(12%)
**TB diagnosis, *n* (%)**										
Observations	8766	(78%)	16,667	(87%)	21,957	(80%)	12,502	(84%)	59,892	(84%)
Males	861	(31%)	1779	(31%)	1864	(26%)	848	(20%)	5352	(27%)
Females	1319	(22%)	2263	(21%)	1946	(13%)	870	(10%)	6398	(16%)
**Weight (kg), med (IQR)**										
Observations	7293	(65%)	14,086	(74%)	19,471	(71%)	9783	(66%)	50,633	(70%)
Males	61	(54–68)	60	(54–68)	62	(55–69)	63	(57–72)	61	(55–69)
Females	60	(52–69)	61	(53–71)	64	(55–76)	68	(58–81)	63	(54–75)
**Pregnant females, *n* (%)**	523	(8%)	915	(8%)	1620	(10%)	1449	(16%)	4507	(10%)

Men started ART with more severe disease progression than women: compared with women, men had lower median CD4 counts (83 vs. 108 cells/µL in 2004–2006, 184 vs. 267 cells/µL in 2013–2015), were more likely to start ART with a CD4 count <50 cells/µL (25% vs. 15%), less likely to initiate treatment with a CD4 count ≥350 cells/µL (12% vs. 28% in 2013–2015) and more likely to have WHO Stage III or IV at ART start. Twice the proportion of men compared with women initiating ART in 2013–2015 had TB. The fraction of women who were pregnant at ART initiation was twice as high in the last quartile of enrolment as in the first (16% vs. 8%). Patients with and without IDs were similar in baseline characteristics (Supplementary Table 1).

Patients were followed for 350,376 pyrs, with a median (IQR) follow-up of 4.48 (2.42–7.03) pyrs. The median follow-up time was 1.28 (0.25–3.47) pyrs among patients who died and 4.93 (3.08–7.41) pyrs among patients who were alive at study end. Women were followed for longer than men: median (IQR) 4.61 (2.55-7.17) vs. 4.24 (2.14-6.71) pyrs. At analysis closure, 10,796 (15%) patients had died: 21% (*n* = 4891) of male and 12% (*n* = 5905) of female patients. The crude mortality rate was 3.08 (95% CI 3.02–3.14)/100 pyrs; the rate among men was nearly double the rate in women (4.50 [95% CI 4.38–4.63] vs. 2.44 [95% CI 2.38–2.51]/100 pyrs).

**Figure 1.**
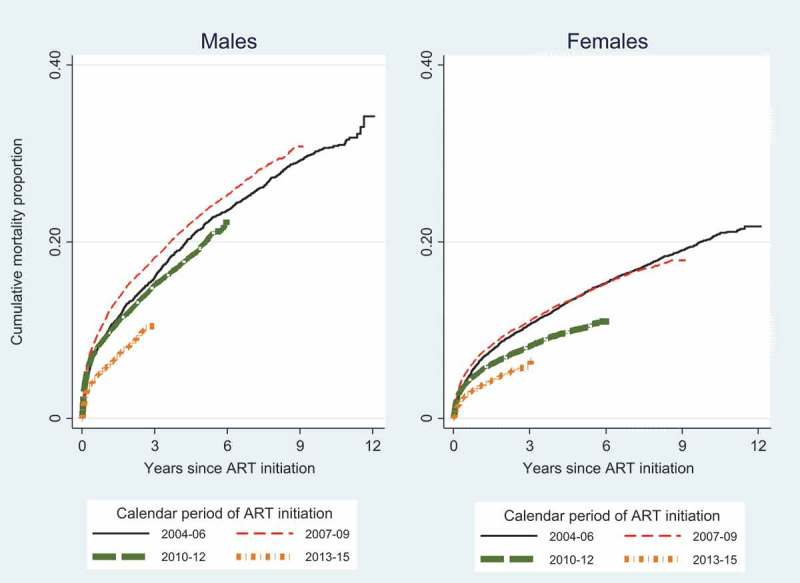
Cumulative mortality by calendar period of ART initiation and duration, by gender.

Twelve years after ART initiation, cumulative mortality was 23.9% (95% CI 22.8–25.1%), and higher among men than women (33.4% vs. 19.4%) (Supplementary Table 2). Mortality peaked in patients initiating treatment in 2007–2009 (Figure 1). In subsequent calendar periods of ART initiation, mortality was lower at all durations. Mortality was higher in men than women at all durations and in all calendar periods of ART initiation.

Results of the SMR analysis are shown in Table 2. Observed mortality was higher than HIV-negative age-standardized mortality in both men and women, particularly in the first year since ART initiation. Observed mortality rates dropped with duration since ART initiation, and by four years, were close to those in the HIV-negative population. Observed mortality rates also declined with increasing CD4 count at ART initiation, and by four years, SMRs were similar for all baseline CD4 strata.

**Table 2.** Observed mortality rates (per 100 person-years) and standardized mortality ratios (SMRs)^a^ by gender, duration since ART initiation and CD4 count at ART initiation^b^.

	Males				Females			
	Duration since ART initiation (months)				Duration since ART initiation (months)			
	**0–12**	**12–24**	**24–48**	**>48**	**0–12**	**12–24**	**24–48**	**>48**
**CD4 count <50 cells/µL**								
Observed mortality (crude)	19.54	5.55	4.07	2.69	16.93	4.26	2.55	1.66
Observed deaths	865	215	265	242	961	217	227	233
HIV-negative mortality (age standardized)	0.78	0.82	0.89	1.05	0.36	0.38	0.41	0.48
SMR	24.98	6.76	4.57	2.57	46.71	11.36	6.27	3.46
SMR 95% CI	23.31–26.64	5.86–7.67	4.02–5.12	2.25–2.9	43.76–49.67	9.84–12.87	5.46–7.09	3.02–3.91
**CD4 count 50–199 cells/µL**								
Observed mortality (crude)	8.65	3.67	3.17	2.84	5.33	2.08	1.65	1.44
Observed deaths	871	336	494	545	1088	399	566	697
HIV-negative mortality (age standardized)	0.86	0.90	0.97	1.12	0.38	0.40	0.43	0.50
SMR	10.01	4.06	3.27	2.53	13.91	5.21	3.83	2.90
SMR 95% CI	9.35–10.68	3.62–4.49	2.98–3.56	2.32–2.74	13.09–14.74	4.7–5.72	3.52–4.15	2.69–3.12
**CD4 count ≥200 cells/µL**								
Observed mortality (crude)	4.01	2.65	2.04	2.11	2.46	1.27	1.15	1.13
Observed deaths	191	104	115	81	357	154	202	143
HIV-negative mortality (age standardized)	0.85	0.88	0.95	1.10	0.37	0.38	0.42	0.47
SMR	4.73	3.01	2.15	1.92	6.74	3.31	2.76	2.39
SMR 95% CI	4.06–5.4	2.43–3.58	1.76–2.54	1.5–2.34	6.04–7.44	2.79–3.83	2.38–3.14	2.0–2.78

^a^SMR: The number of observed deaths divided by the number of deaths expected if all patients were HIV negative.

^b^Excludes 1228 patients who had no CD4 count at ART initiation.

In univariate analysis, the risk of death was higher among males and older patients, and among those with lower CD4 counts, higher WHO stage, TB, lower weight and earlier calendar period of ART initiation (Table 3). In multivariable analysis, most associations persisted but were attenuated, with increased risk among patients who were male (aHR 1.41, 95% CI 1.36–1.47) and older (aHR 3.14, 95% CI 2.68–3.67, patients 65 and older vs. 16–29 years), with lower CD4 counts (aHR 0.40, 95% CI 0.38–0.44: CD ≥200 vs. <50 cells/µL) and higher WHO stage (aHR 2.09, 95% CI 1.93–2.26, WHO stage IV vs. I and II). For every 10 kg increase in weight at ART initiation, there was a 23% decrease in mortality risk (aHR 0.77, 95% CI 0.76–0.79). There was a modest decline in the risk of death in patients initiating ART in 2013–2015 compared with 2004–2006 (aHR 0.85, 95% CI 0.78–0.92). After adjustment for other variables, a TB diagnosis at ART initiation was associated with a reduction in mortality risk (aHR 0.93, 95% CI 0.88–0.99).

**Table 3.** Crude and adjusted^a^ associations with mortality.

	Mortality	
	Univariate HR (95% CI)	Multivariable aHR (95% CI)
Gender, male	1.80 (1.74–1.87)	1.41 (1.36–1.47)
Age (years)		
16–29	1	1
30–34	1.14 (1.07–1.21)	1.04 (0.98–1.11)
35–39	1.28 (1.20–1.35)	1.12 (1.05–1.19)
40–44	1.46 (1.37–1.56)	1.28 (1.20–1.37)
45–49	1.60 (1.49–1.72)	1.40 (1.30–1.51)
50–54	1.93 (1.79–2.11)	1.71 (1.57–1.86)
55–59	2.38 (2.18–2.66)	2.10 (1.90–2.33)
60–64	3.02 (2.68–3.53)	2.63 (2.28–3.04)
65+	3.87 (3.35–4.55)	3.14 (2.68–3.67)
CD4 count (cells/µL)		
<50	1	1
50–99	0.69 (0.65–0.73)	0.74 (0.70–0.79)
100–199	0.45 (0.44–0.49)	0.56 (0.54–0.60)
≥200	0.26 (0.25–0.29)	0.40 (0.38–0.44)
WHO stage		
I and II	1	1
III	2.24 (2.12–2.37)	1.55 (1.46–1.66)
IV	3.26 (3.04–3.48)	2.09 (1.93–2.26)
TB diagnosis	1.62 (1.55–1.70)	0.93 (0.88–0.99)
Weight, 10 kg	0.71 (0.69–0.72)	0.77 (0.76–0.79)
Year ART initiated		
2004–2006	1	1
2007–2009	1.00 (0.95–1.05)	1.03 (0.98–1.14)
2010–2012	0.73 (0.69–0.77)	0.98 (0.92–1.03)
2013–2015	0.47 (0.44–0.51)	0.85 (0.78–0.92)
Site of ART initiation		
Cohort 1	1	1
Cohort 2	2.07 (1.88–2.28)	1.91 (1.73–2.11)
Cohort 3	1.38 (1.25–1.53)	1.36 (1.22–1.51)
Cohort 4	1.72 (1.56–1.90)	1.46 (1.31–1.62)
Cohort 5	2.35 (2.09–2.64)	1.83 (1.62–2.07)

^a^After multiple imputation.

Overall, 54,045 (74%) patients had VL measures in their last year of follow-up (Table 4). Among patients with VLs, 86% (*n* = 46,675) were virally suppressed, with slightly higher proportions among women compared to men in all calendar periods of ART initiation.

**Table 4.** Completeness of VL measures and number (percentage) of patients virally suppressed^a^ in their last year of follow-up, by gender and calendar period of ART initiation.

	Calendar period of ART initiation									
	2004–2006		2007–2009		2010–2012		2013–2015		Total	
**Patients (*n*)**										
All	11,243		19,148		27,516		14,905		72,812	
Males	3537		6531		8974		4745		23,787	
Females	7706		12,617		18,542		10,160		49,025	
**VL measures, *n* (%)**										
All	8594	76%	14,531	76%	20,337	74%	10,583	71%	54,045	74%
Males	2593	73%	4791	73%	6356	71%	3279	69%	17,019	72%
Females	6001	78%	9740	77%	13,981	75%	7304	72%	37,026	76%
**Virally suppressed^b^, *n* (%)**										
All	7267	85%	12,056	83%	17,946	88%	9406	89%	46,675	86%
Males	2136	82%	3804	79%	5413	85%	2854	87%	14,207	83%
Females	5131	86%	8252	85%	12,533	90%	6552	90%	32,468	88%

^a^Viral suppression: viral load <1000 copies/mL.

^b^Of those with viral load measures, percentage with viral load <1000 copies/mL.

## Discussion

In this cohort of adults starting ART in South Africa with near-complete ascertainment of deaths, three-quarters of patients were alive twelve years after initiation of treatment. Our findings provide reassurance that the national ART programme is functioning effectively. Despite increased enrolment, mortality was remarkably stable across calendar periods and with duration on treatment. With increasing duration on ART, SMRs and absolute mortality differences compared to HIV-negative individuals declined. Markers of disease severity at ART initiation improved, suggesting improved coverage. High levels of viral suppression were sustained over time, but gender disparities in ART initiation and mortality persisted.

Despite a delayed start, the South African ART programme has expanded ART services enormously since its inception in 2004, increasing enrolment each successive year. Concurrently, the entire national health system has undergone major restructuring to a district health system implementing primary healthcare, placing additional strain on an already heavily burdened health system [39]. Despite these challenges, 74% of patients were still alive twelve years after starting treatment, suggesting that early fears of “antiretroviral anarchy” in Africa [40] were unfounded.

Numerous studies over the past ten years have addressed the complex issue of mortality estimation, particularly in developing countries [41–46]. Many countries with large, rapidly expanding ART programmes lack functioning vital registration systems, and mortality ascertainment from standard patients’ record systems may misclassify a high proportion of deaths as LTF [12,41]. To correct for mortality among patients LTF, different approaches have been used including tracing studies [47–49] and the application of a nomogram [44]. However, using different methods of mortality ascertainment can impact on mortality estimates. In our study, linkage of patient records to the well-functioning NPR has ensured that all patients have known outcomes at analysis closure, including those who were LTF or transferred. It is thus unsurprising that our mortality estimates are higher than long-term reports from other large national ART programmes which rely on passive mortality ascertainment and report high rates of LTF. For example, among 226,030 patients who started ART in the Botswana national programme between 2002 and 2013, the reported mortality rate was lower than in our study (2.06/100 vs. 3.10/100 pyrs), while the rate of LTF was high (12.47/100 pyrs) [50]. Studies which compare and combine mortality data must thus consider the possible impact of differential mortality ascertainment on their estimates.

Mortality estimates from ART programmes also rarely include a comparison with HIV-negative mortality rates. Using age-standardization, we were able to compare observed mortality with rates in the HIV-negative population. Observed mortality was far higher than in the HIV-negative population, particularly in the first year after ART initiation. By four years after ART initiation, SMRs were similar in all CD4 strata, suggesting that baseline immunologic status no longer impacted mortality rates substantially. Due to far higher background mortality rates in men compared with women, female SMRs were higher than male, even though all-cause mortality rates in men were higher than those in women. SMRs declined towards 1 as ART duration increased, suggesting an increasing “normalization” of mortality in ART patients with duration on treatment [51,52]. Our results highlight the need to assess and compare mortality estimates in ART programmes in the context of background mortality.

The steady increase in median CD4 count in successive calendar periods of ART initiation reflects improved coverage, as the programme progressed from initiating only the sickest patients. In a previous study, the median CD4 count at ART initiation increased worldwide between 2000 and 2009 but remained <200 cells/µL in low- and middle-income countries including South Africa [53]. We demonstrated a sustained increase in successive calendar periods, reaching 242 cells/µL in patients enrolled in 2013–2015, nearly 2.5 times that in the earliest enrolled patients. With the introduction of universal eligibility for ART, baseline CD4 counts should continue to increase.

However, it appears that increasing CD4 count at enrolment did not always result in decreasing first-year mortality. In these cohorts, after adjusting for baseline CD4 count, first-year mortality continued to rise over the first few years of ART, only decreasing after 2009 (Supplementary Table 3). It is likely that the rapid scale-up of the programme and the contribution of different cohorts contributed to the increasing mortality up to 2009. In our analysis, we did not focus on differences between sites of ART initiation; however, there were differences (numbers enrolled and mortality estimates) between hospital and primary care cohorts, and between urban and rural cohorts. It is plausible that the differential contribution of cohorts over time partially explains the failure to see an initial decline in early mortality.

The apparently protective effect of TB at ART initiation has been previously observed [8] and may be related to the close case-holding that happens in coinfected patients who need to attend the clinic far more regularly than patients who are not coinfected. It could also be related to the fact that in patients with TB at ART initiation, the TB is identified and treated, whereas unrecognized TB is postulated to be a large contributor to early mortality.

As services expand further, high levels of viral suppression must be maintained. The national ART guidelines recommend VL testing as the “preferred approach for monitoring ART success and diagnosing treatment failure” [34]. Despite increasing patient numbers, 74% of all patients in these cohorts had a VL measured in their last year of follow-up. It is reassuring that 86% of these patients were virally suppressed, close to the UNAIDS target of 90% suppression. This compares well with national and global figures. In 2015, 78.4% of patients on ART tested nationwide had undetectable VLs, and at a threshold of 1000 copies/mL, this fraction would be 81.7% [2]. Ongoing monitoring is required to ensure that high levels of suppression are sustained.

We found evidence that programmes to prevent mother-to-child transmission of HIV (PMTCT) may have increased ART initiation in pregnant women. PMTCT guidelines have evolved over the years, increasing pregnant women’s access to HIV testing and ART. Option B, introduced in 2013 [54], recommended ART for the mother from 14-weeks gestation through to birth or the end of breastfeeding, while Option B+, introduced in 2015 [34], advocated lifelong ART for the mother. National ART guidelines also recommend routine HIV testing as part of clinical care and screening for pregnant and breastfeeding women: testing every three months throughout pregnancy, at labour/delivery, at the 6-week immunization visit, and every three months throughout breastfeeding [34]. Women who choose not to be tested must be provided with “post-refusal” counselling and then offered testing again at all subsequent visits. We found that these guidelines increased ART initiation among pregnant women: the proportion of women who were pregnant at ART initiation doubled, with most of the increase in patients enrolled in 2013–2015. During the same time period, the proportion of women starting ART with CD4 ≥350 cells/µL was twice that among men.

Unfortunately, South African ART programmes have had less success in ensuring equitable access to ART for men. Despite overwhelming evidence of men’s poorer access to HIV testing [25,27] and ART initiation [23,24,26,55,56], men still started ART at older ages and with more advanced HIV disease than women in all calendar periods. Furthermore, the proportion of men starting ART did not increase over twelve years. This is in line with findings from a large national study by the NHLS which analysed nearly 4 million CD4 count and VL measurements from 2012 to estimate progress towards the UNAIDS 90:90:90 targets []. At each stage of the ART cascade, men had poorer engagement than women [57]. With the implementation of Option B+, it is possible that such gender disparities in access to ART may increase, as has been reported in Malawi [58] and Mozambique [3]. National campaigns are urgently needed to increase testing and earlier ART initiation among men.

This study was strengthened by the large sample size, the duration of follow-up, the comparison with HIV-negative mortality and, in particular, by linkage to the NPR allowing accurate ascertainment of mortality. Interpretation of these results has several limitations. First, these data from predominantly urban research cohorts may not represent all public sector ART cohorts; hence, these results may not be generalizable in all settings. Second, as these are observational data, a proportion of data were missing for most baseline variables, a limitation we addressed through the use of multiple imputation. Third, notwithstanding the similar baseline characteristics reported between patients with and without IDs, if these patient groups differed in unmeasured ways, our findings could be biased. Similarly, VL assessments were not available for a quarter of patients in their last year of follow-up, which might bias our assessment of viral suppression. However, in analysis restricted to patients who had VLs measured at any timepoint, there was little difference in the proportions virally suppressed at their last VL measurement, comparing those with and without a VL in their last year of follow-up (86% vs. 83%) (Supplementary Table 4). Finally, our available measures of disease severity used to adjust our analyses might not fully capture the improving clinical status at ART initiation over time.

In conclusion, the South African ART programme has demonstrated a remarkable ability to initiate and manage patients successfully over twelve years, despite rapidly expanding patient numbers. As the country scales up services to treat the roughly 3 million individuals still requiring ART in 2017, testing and initiating men on ART must be a national priority.

## Notes

To access the supplementary material to this article please see Supplementary Files under Article Tools online.
